# Incidence of invasive infections with Group B streptococcus in adults in Norway 1996–2019: a nationwide registry-based case–control study

**DOI:** 10.1007/s15010-024-02210-3

**Published:** 2024-03-14

**Authors:** Elise Uggen, Camilla Olaisen, Randi Valsø Lyng, Gunnar Skov Simonsen, Roar Magne Bævre-Jensen, Frode Width Gran, Bjørn Olav Åsvold, Tom Ivar Lund Nilsen, Jan Kristian Damås, Jan Egil Afset

**Affiliations:** 1 Faculty of Medicine and Health Sciences, Norwegian University of Science and Technology, Trondheim, Norway; 2 Mid-Norway Centre of Sepsis Research, Trondheim, Norway; 3 Department of Medical Microbiology, St. Olavs Hospital, Trondheim University Hospital, Trondheim, Norway; 4 Department of Microbiology and Infection Control, University Hospital of North Norway, Tromsø, Norway; 5 Research Group for Host-Microbee Interaction, Faculty of Health Sciences, UiT – The Arctic University of Norway, Tromsø, Norway; 6 Department of Medical Microbiology, Vestre Viken Hospital Trust, Drammen, Norway; 7 HUNT Center for Molecular and Clinical Epidemiology, Department of Public Health and Nursing, NTNU, Trondheim, Norway; 8 HUNT Research Center, Department of Public Health and Nursing, NTNU, Levanger, Norway; 9 Department of Endocrinology, Clinic of Medicine, St. Olavs Hospital, Trondheim University Hospital, Trondheim, Norway; 10 Department of Public Health and Nursing, Norwegian University of Science and Technology, Trondheim, Norway; 11 Clinic of Anaesthesia and Intensive Care, St Olavs Hospital Trondheim University Hospital, Trondheim, Norway; 12 Department of Clinical and Molecular Medicine, Faculty of Medicine and Health Sciences, Norwegian University of Science and Technology, Trondheim, Norway; 13 Department of Infectious Diseases, Clinic of Medicine, St. Olavs Hospital, Trondheim University Hospital, Trondheim, Norway

**Keywords:** Group B streptococcus, GBS, *Streptococcus agalactiae*, Invasive disease, Incidence, Risk factors

## Abstract

**Purpose:**

Group B streptococcus (GBS) colonizes the gastrointestinal and vaginal mucosa in healthy adults, but has also become an increasing cause of invasive infection. The aims of this study were to describe the incidence and factors associated with the occurrence of invasive GBS disease in adults in Norway.

**Methods:**

We performed a nationwide retrospective case–control study of invasive GBS infections during 1996–2019, with two control groups; invasive Group A streptococcal disease (GAS) to control for changes in surveillance and diagnostics, and a second representing the general population.

**Results:**

A total of 3710 GBS episodes were identified. The age-standardized incidence rate increased steadily from 1.10 (95% CI 0.80–1.50) in 1996 to 6.70 (95% CI 5.90–7.50) per 100,000 person-years in 2019. The incidence rate had an average annual increase of 6.44% (95% CI 5.12–7.78). Incidence rates of GAS varied considerably, and there was no evidence of a consistent change over the study period. GBS incidence was highest among adults > 60 years of age. Cardiovascular disease, cancer, and diabetes were the most common comorbid conditions. There was a shift in the distribution of capsular serotypes from three dominant types to more equal distribution among the six most common serotypes.

**Conclusions:**

The incidence of invasive GBS disease in adults increased significantly from 1996 to 2019. The increasing age of the population with accompanying underlying comorbid conditions might contribute to the increasing burden of invasive GBS disease. Interestingly, type 1 diabetes was also associated with the occurrence of invasive GBS disease.

**Supplementary Information:**

The online version contains supplementary material available at 10.1007/s15010-024-02210-3.

## Introduction

*Streptococcus agalactiae* (group B streptococcus, GBS) is best known as a leading cause of neonatal sepsis but also constitutes an important cause of infections in adults [1, 2]. While incidence rates among newborns are stable [3], there has been an increase in invasive GBS infections in adults [4–11]. The rates of invasive disease among older adults have doubled over the past decade in several countries [3, 4, 7, 12].

Risk factors for invasive GBS disease in adults include old age and chronic medical conditions such as diabetes, obesity, and cancer [4, 7, 13, 14]. Some authors propose that the increasing prevalence of underlying medical conditions is the most plausible explanation for the increasing incidence of invasive GBS infection in adults [4].

Traditionally, classification of GBS for epidemiological purposes is based on serotyping of ten (﻿Ia, Ib, II–IX) different capsular polysaccharide (CPS) types. Typing of GBS is of value for the ongoing efforts to develop a vaccine against invasive GBS infection with focus on infections in the newborn [14]. Such a vaccine might also be useful for prevention of invasive GBS infections in adults. A better understanding of the epidemiology of GBS infections including risk groups that might benefit from a vaccine is important. As well as the distribution of potential vaccine targets, such as CPS and other surface protein types including the alpha like proteins (Alps) would be valuable for developing a GBS vaccine and planning of vaccination strategy [15].

The aim of this study is to provide an overview of the epidemiology of invasive GBS infections in adults in Norway from 1996 to 2019, focusing on trends in incidence rates and factors associated with the occurrence of invasive GBS disease.

## Methods

Invasive GBS infection has been notifiable to the Norwegian Surveillance System for Communicable Diseases (MSIS) since 1986. Throughout this period St. Olavs hospital has received GBS isolates for typing from other laboratories, and since 2006 as a national reference laboratory. For this study we first identified all patients ≥ 18 years of age where the national reference laboratory for GBS had received GBS cultured from patients with invasive infection during the period 1996–2019 (Supplementary Fig. 1). This list was then complemented with episodes of GBS notified to MSIS. For the resulting list of study participants (*n* = 3537), we requested data from the Norwegian Patient Registry (NPR).

We conducted the study as a retrospective study with two control groups. The first control group consisted of all adult patients ≥ 18 years of age with reported invasive *Streptococcus pyogenes* (Group A streptococcus, GAS) infection, also notifiable to MSIS. Since individual data is only available in NPR from 2008 onwards, we were not able to assess risk factors for infectious episodes during the period 1996–2007. For this reason, we were left with 2324 individuals with GBS infection and 2003 individuals with GAS infection. Comorbid conditions during the period 2008–2019 were identified in NPR for 2311 GBS patients and 1984 GAS patients. A second control group of general population controls was randomly selected from the National Population Registry among inhabitants who were alive at 1st January 2008. We selected four individuals for each of the 2311 GBS cases, matched on year of birth, thus constituting 9244 controls.

We collected data from NPR on duration of hospital stay, age, sex and selected ICD-10 diagnoses for comorbid conditions potentially relevant for GBS infection (Table 1). We included ICD-10 codes for conditions registered prior to the infectious episode ICD-10 codes were extracted from discharge diagnoses after hospital stays, but also from contract specialists that treat conditions that do not require hospitalizations such as in outpatient clinics. For population controls, diagnosis codes were extracted prior to the hospitalization date of the matched GBS patient. In addition, we received data on the annual number of patients who had blood cultures taken during the study period from seven different hospitals.

**Table 1:** Clinical characteristics of patients with invasive Group B streptococcus infection (GBS) and two control groups: patients with invasive group A streptococcus (GAS) infection and randomly selected general population controls 1996–2019

Group	GBS	GAS	General population controls
All patients, *n* (%)^a^	3537 (100)	4064 (100)	9244 (100)
Male sex, *n* (%)^b^	1701 (49.7)	2023 (49.8)	3491 (46.7)
Age, median (IQR)	68 (53–79)	62 (42–76)	70 (56–80)
Individuals with recurrent disease, *n*(%)^c﻿^	144 (4.1)	55 (1.4)	NA
Recurrent episodes, *n*	173	59	NA
Days hospitalized, median (IQR)^d^	7 (4–15)	8 (4–16)	NA
Sample type^e^			
Blood culture, *n* (%)	2956 (94.6)	3567 (87.0)	NA
Joint, *n* (%)	56 (1.8)	55 (1.3)	NA
Cerebrospinal fluid, *n* (%)	25 (0.8)	33 (0.8)	NA
Other, *n* (%)	87 (2.8)	443 (10.8)	NA
Comorbidity			
Cancer, *n* (%)^g^	592 (25.5)	257 (12.8)	821 (8.9)
Type 1 diabetes, *n* (%)	242 (10.4)	84 (4.2)	147 (1.6)
Type 2 diabetes, *n* (%)	501 (21.6)	214 (10.7)	567 (6.1)
Iron deficiency anemia, *n* (%)	130 (5.6)	79 (3.9)	162 (1.8)
Neurological disease, *n* (%)^h^	176 (7.6)	95 (4.7)	310 (3.4)
Cardiovascular disease, *n* (%)^i^	1028 (44.2)	585 (29.2)	2115 (22.9)
Respiratory disease, *n* (%)^j^	257 (11.1)	185 (9.2)	660 (7.1)
Gastrointestinal disease, *n* (%)^k^	110 (4.7)	71 (3.5)	120 (1.3)
Rheumatic disease, *n* (%)^l^	182 (7.8)	137 (6.8)	394 (4.3)
Urogenital disease, *n* (%)^m^	370 (15.9)	187 (9.3)	368 (4.0)
Skin and soft tissue disease, *n* (%)^n^	77 (3.3)	59 (2.9)	149 (1.6)

IQR: inter quartile range

^a^Available data on age in 3523 (99.9%) of GBS patients and 4064 (100%) of GAS patients

^b^Available data on sex in 3420 (96.7%) GBS patients, 4064 (100%) GAS patients and 7472 (80%) general population controls

^c^Recurrent disease was defined as a positive GBS sample from a normally sterile body site > 30 days after the last episode; numbers correspond to unique individuals with a first episode who experience recurrent disease

^d^Avaliable from 2008 to 2019 from the Norwegian Patient Registry (NPR) based on admissions identified from sample date for 2311 GBS cases and 1984 GAS cases

^e^As registered in the Norwegian Surveillance System for Communicable Diseases (MSIS), available for all notified cases, 3124 GBS cases (84.4%) and 4098 (99.4%) GAS cases

^f^Avaliable from 2008 to 2019 from the Norwegian Patient Registry (NPR) for 2324 GBS cases and 2003 GAS cases

^g^Included international classification of diseases codes (ICD-10): C15, C16, C18, C21, C24, C25, C34, C43, C50, C53 C54,C56, C56, C61, C62, C67 C71 C81, C82, C83, C90, C91, C92

^h^Included ICD-10 codes: G35, G20, G40, G62 G80

^i^Included ICD-10 codes: I10, I20, I21, I25, I42, I50, I63

^j^Included ICD-10 codes: J44, J45, J84

^k^Included ICD-10 codes: K50, K51, K70, K72, K74, K75

^l^Included ICD-10 codes: M05, M06, M13, M31, M32, M35, M45

^m^Included ICD-10 codes: N04, N05, N13, N18, N19

^n^Included ICD-10 codes: L23, L24, L40

We defined an episode of invasive GBS disease as GBS isolated from a normally sterile body site. The infection was classified as recurrent if GBS was isolated again ≥ 30 days after a prior episode. We analysed all the GBS isolates received at the national reference laboratory for CPS-type (Ia, Ib, II–IX), alp genes (*bca*, *alp1*/*epsilon*, *alp2/3*, *alp4*, *rib*), and the *bac* gene encoding the C beta protein, by polymerase chain reaction (PCR) [16].

We calculated crude and standardized incidence rates of a first invasive GBS- and GAS-disease per 100,000 person-years, using data from Statistics Norway (SSB) on number of residents in Norway at the beginning of each calendar year as the denominator. Rates were standardized according to the age distribution of the world standard population (Segi). Incidence rates were stratified according to sex, age group, and time period. We estimated the temporal trend in annual incidence rates (1996–2019) by fitting a least-squares regression model to the log-transformed age-standardized rates, weighted by the inverse of their variance. We used Poisson regression to estimate incidence rate ratios (IRR) between age groups and sex. Logistic regression estimated odds ratios of comorbid conditions comparing GBS cases with GAS controls, and conditional logistic regression comparing GBS cases with general population controls adjusting for the matched design. We performed multiple imputation for missing data on sex (*n* = 1772 out of 9244 individuals) in the general population control group. Seven patients with missing data on age were not imputed, and thus excluded from the adjusted analysis. We used 10 imputed data sets in the estimation. Precision of all estimated associations was assessed using 95% confidence intervals (CI). All analyses were conducted using STATA version 17sk. Figures were created using GraphPad Prism 9.4.1 (GraphPad Prism Software, San Diego, CA, USA).

The study was approved by The Regional Committee for Medical and Health Research Ethics (REK-Midt, application number: 7175). Data protection impact assessment was approved by the head, Clinic of Laboratory Medicine, St. Olav´s University Hospital. Data from national health registries were received after approval from each registry, without consent from patients.

## Results

### Clinical characteristics

A total of 3710 invasive GBS infection episodes occurred from 1996 through 2019 in 3537 unique adult patients. The control group with invasive GAS infection included 4123 episodes in 4064 individuals. Among patients with GBS disease, 49.7% were male (Table 1). The median age of GBS patients was 68 years (interquartile range, IQR 53–79) compared to 62 years (IQR 42–76) for GAS patients. Bacterial isolates were mostly from blood (GBS 94.6%; GAS 87.0%).

### Incidence

**Fig. 1:**
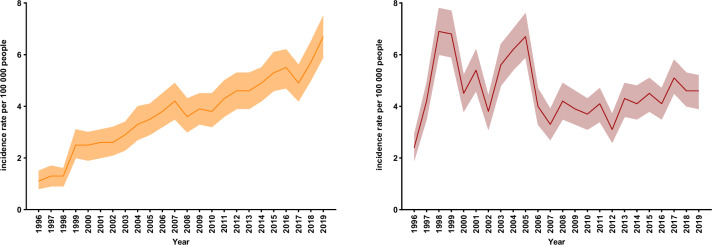
Age standardized incidence rates of **A** invasive group B streptococcal disease and **B** invasive group A streptococcal disease per 100,000 with 95% CI

The age-standardized incidence rate of invasive GBS disease increased from 1.10 (95% CI 0.80–1.50) per 100,000 person-years in 1996 to 6.70 (95% CI 5.90–7.50) per 100,000 person-years in 2019 (Fig. 1 and Supplementary Table 1). The incidence rate increased by 0.19 (95% CI 0.16–0.22) per 100,000 per year on average, from 1996 to 2019, corresponding to an annual increase of 6.44% (95% CI 5.12–7.78). The incidence rates of invasive GAS disease varied considerably from year to year. There was an average yearly increase of 0.4% (95% CI -1.17–1.98), but no evidence for a consistent change over the study period.

**Fig. 2:**
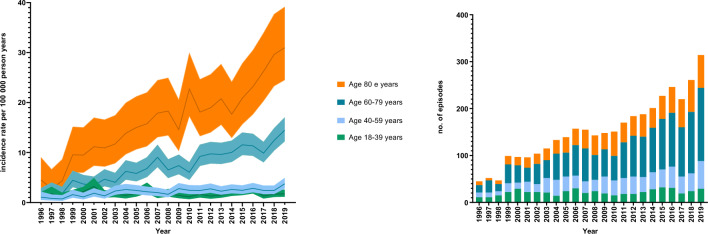
Incidence of invasive group B streptococcal disease in adults 1996–2019 by age group presented as incidence rate (**A**) and number of infection episodes (**B**)

The incidence rate of invasive GBS disease increased most in older age groups and was 14.45 (95% CI 12.27–17.01) and 30.97 (95% CI 24.50–39.15) per 100,000 person-years in patients 60–79 years and those 80 years or older, respectively in 2019 (Fig. 2A and Supplementary Table 2). The highest number of patients was observed in the age group 60–79 years (Fig. 2B). When stratified by sex, the incidence rate of invasive GBS disease in men was 6.79 (95% CI 6.24–7.40) per 100,000 person-years in the period 2016–2019, compared to 5.41 (95% CI 4.95–5.94) per 100,000 person-years in women in the same period. Incidence rate ratios (IRR) in men compared to women changed from 0.70 (95% CI 0.55–0.88) at the beginning of the study period to 1.26 (95% CI 1.11–1.43) in 2016–2019. In the period 2016–2019, the IRR comparing men and women spanned from 0.32 (95% CI 0.20–0.51) in patients 18–39 years to 1.88 (95% CI 1.46–2.41) in those ≥ 80 years, respectively (Supplementary Table 3).

### Blood cultures

We received data on number of blood cultures collected from six hospitals. The number of patients where blood culture had been collected increased by 138% (varied from 59 to 316% increase between hospitals), from 8483 patients in 1996 to 20,203 patients in 2019 (Supplementary Fig. 2 and Supplementary Table 4). During the same period the population in Norway aged ≥ 18 years increased by 25.3% (Statistics Norway).

### Comorbidity

The comorbid conditions with the highest proportions in GBS patients were cardiovascular disease (44.2%), diabetes (32.0%), and cancer (25.5%) (Table 1). After adjustment for age and gender, the odds ratio (OR) for type 2 diabetes was 2.07 (95% CI 1.74–2.47) in GBS patients compared to GAS patients, and 4.15 (95% CI 3.46–4.97) compared with general population controls (Table 2). For type 1 diabetes the adjusted OR was 2.58 (95% CI 1.99–3.37) compared to GAS, and 6.98 (5.29–9.22) compared to general population controls. The proportion of patients with systemic GBS infection who also had type 2 diabetes increased from 16.63% (95% CI 14.39–19.15) in the period 2008–2013 to 24.93% (95% CI 22.71–27.28) in the period 2014–2019 (Table 2). We also found a significant rise in proportion of GBS patients with cancer, cardiovascular and neurological diseases from 2008–2013 to 2014–2019 (Supplementary Table 5).

**Table 2:** Odds ratio for type 1 and type 2 diabetes in patients with invasive group B streptococcal disease (GBS) versus invasive group A streptococcal disease (GAS) and general population controls

Type of diabetes and time period	No. of GBS cases	Proportion GBS patients with diabetes % (95% CI)	OR (95% CI)			
			Crude GBS/GAS	Adjusted GBS/GAS^a^	Crude GBS/General population	Adjusted GBS/General population^a^
Type 2 Diabetes	501	21.6 (19.9–23.3)	2.30	2.07 (1.74–2.47)	4.54	4.15 (3.46–4.97)
2008–2013	157	16.6 (14.4–19.2)	2.06	1.90 (1.42–2.55)	4.80	4.80 (3.46–6.67)
2014–2019	344	24.9 (22.7–27.3)	2.40	2.14 (1.72–2.67)	4.41	3.88 (3.12–4.82)
Type 1 Diabetes	242	10.3 (9.2–11.7)	2.66	2.58 (1.99–3.37)	7.25	6.98 (5.29–9.22)
2008–2013	85	9.0 (7.3–11.0)	2.27	2.27 (1.52–3.39)	7.26	7.90 (4.92–12.69)
2014–2019	157	11.4 (9.8–13.2)	2.92	2.77 (1.88–3.22)	7.25	6.51 (4.61–9.18)

^a^Adjusted for age and gender

### Bacterial isolates

**Fig. 3:**
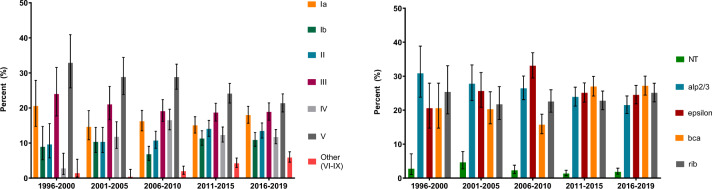
Proportion of **A** capsular serotypes and **B** surface protein genes, with 95% confidence intervals, 5-year periods ^*^last time period only four years

The relative distribution of capsular serotypes changed during the study period (Supplementary Table 6 and Fig. 3A). The three most common CPS types V, Ia and III accounted for 77.4% and 58.1% of the strains in the periods 1996–2001 and 2016–2019, respectively. In contrast, the CPS types Ib, II, IV increased in relative proportion from 22.1% to 36.0% during the same period. Among Alp genes there was a change toward more equal distribution of the four most common Alp-types during the study period (Fig. 3B).

## Discussion

The incidence of invasive GBS infections in adults in Norway increased more than sixfold during the study period from 1996 to 2019. In comparison, the incidence of invasive GAS infections varied considerably during the same period, but without a similar increasing trend. This indicates that changes in surveillance systems alone cannot explain the increased incidence of invasive GBS disease. Although an aging population is likely to contribute to the increasing number of episodes, the incidence rate also increased within the older age groups. This indicates that other factors than age may influence the incidence of invasive GBS infections in the population.

The increase in the incidence of invasive GBS infection found in this study is similar to what has been reported elsewhere [3–5, 7, 12]. The somewhat lower incidence in Norway compared to the U.S could be explained by a lower prevalence of underlying predisposing diseases [4]. However, the observed increase in incidence among older age groups is rather similar in both countries [4]. An aging population with increasing prevalence of comorbid diseases has been pointed out as the main reason for the increasing burden of invasive GBS disease [4, 5]. In our data we see an increase in the proportion of GBS patients with pre-diagnostic comorbidities such as type 2 diabetes, cancer, cardiovascular disease, urogenital disease, and neurological disease from the first period with individual data on comorbidities (2008–2013) compared to the last period (2014–2019). However, since NPR data are available only after 2008, the time window for observing pre-diagnostic comorbidities is shorter for infections occurring before 2013 than those occurring after, possibly leading to a lower proportion with comorbidities in the first compared to the second period. Compared to the two control groups, adjusted for age and gender, the changes in proportions are uncertain, and the increase in predisposing conditions could be similar in cases and controls. Consequently, GBS patients did not have any clear increase in predisposing conditions when compared to GAS patients or to the general population. This could be explained by increasing age followed by multimorbidity in GBS patients, discussed further below.

We found a significant rise in proportion of GBS patients with type 2 diabetes, cancer, cardiovascular and neurological diseases. This aligns with the broader epidemiological trend of age-related and lifestyle-associated diseases becoming more widespread. Consequently, as the adult demographic evolves with conditions that compromise immune function and physiological resilience, the environment for invasive GBS infection might become more pronounced. It is worth noting that while age and chronic medical conditions stand out as prominent risk factors, the interaction between these factors and the diverse mechanisms of GBS pathogenesis remains a subject of ongoing research. Unravelling the relationship between host susceptibility, microbial dynamics, and underlying medical states is important for comprehending the rising incidence of invasive GBS disease, but also for preventive and therapeutic strategies tailored to the vulnerabilities presented by individual patients.

Diabetes has been suggested as one of the leading causes for changes in GBS epidemiology [17]. As diabetes is expected to increase significantly in the coming decades, there is a substantial risk that the incidence of GBS infections will continue to rise [18]. In Norway the prevalence of type 2 diabetes increased from 4.9% in 2009 to 6.1% in 2014 [19]. In this study, the proportion of type 2 diabetes among GBS patients increased during the study period. Both type 1 and type 2 diabetes were overrepresented in GBS patients compared to patients with GAS and general population controls. The pathophysiological mechanisms by which diabetes leads to higher susceptibility to invasive GBS is proposed to be modulation of the immune system and weakening of anatomic barriers [20].

While diabetes is widely recognized as a risk factor for GBS disease it is mainly described in relation to obesity and type 2 diabetes. We found that also type 1 diabetes was strongly associated with invasive GBS disease. The differentiation between type 1 and type 2 diabetes in relation to invasive GBS disease has to our knowledge not been reported before. The fact that both type 1 and 2 diabetes are associated with an increased occurrence of GBS may help elucidate the pathophysiology behind a possible link between diabetes and invasive GBS infection.

The higher incidence of invasive GBS disease in older males in this study is similar to the findings reported by Watkins et al. [4]. However, in contrast to this study they reported higher incidence among males in all age groups. This difference may at least partly be explained by the fact that ages 18–39 most likely were dominated by females with complications during pregnancy and labour in this study. We also found that the number of patients with a blood culture sample increased more than in the general population and this might reflect a concomitant change in disease severity among the patients admitted to hospital during the study period. To our knowledge, there were no major changes in Norway in clinical indication for blood culture or in blood culture methods during this period.

The change in relative distribution of CPS types during the study period corresponds with changes over time reported from the US and Canada [4, 21, 22]. Based on the CPS serotype and Alp type distribution in this study, a hexavalent polysaccharide conjugate vaccine and alp-based vaccine under development would potentially cover 94.1% and 98.2%, respectively, of episodes with invasive GBS disease in adults in Norway [23].

Strengths of this study include the large sample size with nation-wide coverage based on national health registries, the long observation period and inclusion of two control groups. The GAS control group is important for assessing potential changes in the health care system during the study period since it also was mandatorily reported to MSIS throughout the study period. The general population controls are better suited for analysing risk factors for invasive GBS infection. A limitation of the study is that information of risk factors is based on ICD-10 codes reported by the treating physician; thus, we lack data on BMI/obesity which is rarely registered with an ICD code in Norwegian hospitals. Another limitation is that we did not have data on infection focus, only sample type submitted for microbiological analysis, and number of blood cultures sampled per year only from a selection of hospitals. Limitations with the method is that the use of odds ratio for comorbid conditions serves only relative estimates. This means that even if diabetes increased among GBS patients over the study period, it does not necessarily increase relatively to GAS. Also, it does not assess if changes in diabetes occurrence could explain the increase in GBS incidence. Finally, we did not assess whether there was a statistically significant rise in incidence rates within age subgroups due to small sample sizes and low statistical power.

In conclusion, the incidence of invasive GBS disease in adults in Norway increased sixfold during the period from 1996 to 2019. Age, male sex and underlying medical conditions are factors associated with the occurrence of invasive GBS disease. Increasing prevalence of both type 1 and type 2 diabetes might contribute to the increasing burden of invasive GBS disease, and improved prevention and treatment of diabetes may reduce GBS infection risk.

## Supplementary Information

Below is the link to the electronic supplementary material.

Supplementary file1 (PDF 554 KB)

## Data Availability

The data that support the findings in this study are available on reasonable request from the corresponding author.
