# Supplementary material for: Incidence of invasive infections with Group B streptococcus in adults in Norway 1996–2019: a nationwide registry-based case–control study
Source: Infection. 2024 Mar 14;52(5):1745–52. doi: 10.1007/s15010-024-02210-3 (PMC11499455; doi:10.1007/s15010-024-02210-3)
Supplement: Supplementary file 1 — Supplementary file1 (PDF 554 KB) [file 15010_2024_2210_MOESM1_ESM.pdf]

Supplementary information: Incidence of invasive infections with Group B streptococcus in adults in Norway 1996-2019; a nationwide registry-based case-control study. Infection. Elise Uggen, Camilla Olaisen, Randi Valsø Lyng, Gunnar Skov Simonsen, Roar-Magne Bævre-Jensen, Frode Width Gran, Bjørn Olav Åsvold, Tom Ivar Lund Nilsen, Jan Kristian Damås, Jan Egil Afset. **Corresponding author**: Elise Uggen, Faculty of Medicine and Health Sciences, Norwegian University of Science and Technology, 7491 Trondheim, Norway. Email address: eliseu@stud.ntnu.no. Phone number: +47 46885347.

Supplementary Fig. 1: Flowchart showing the data collection process of GBS cases, GAS-cases and general population controls

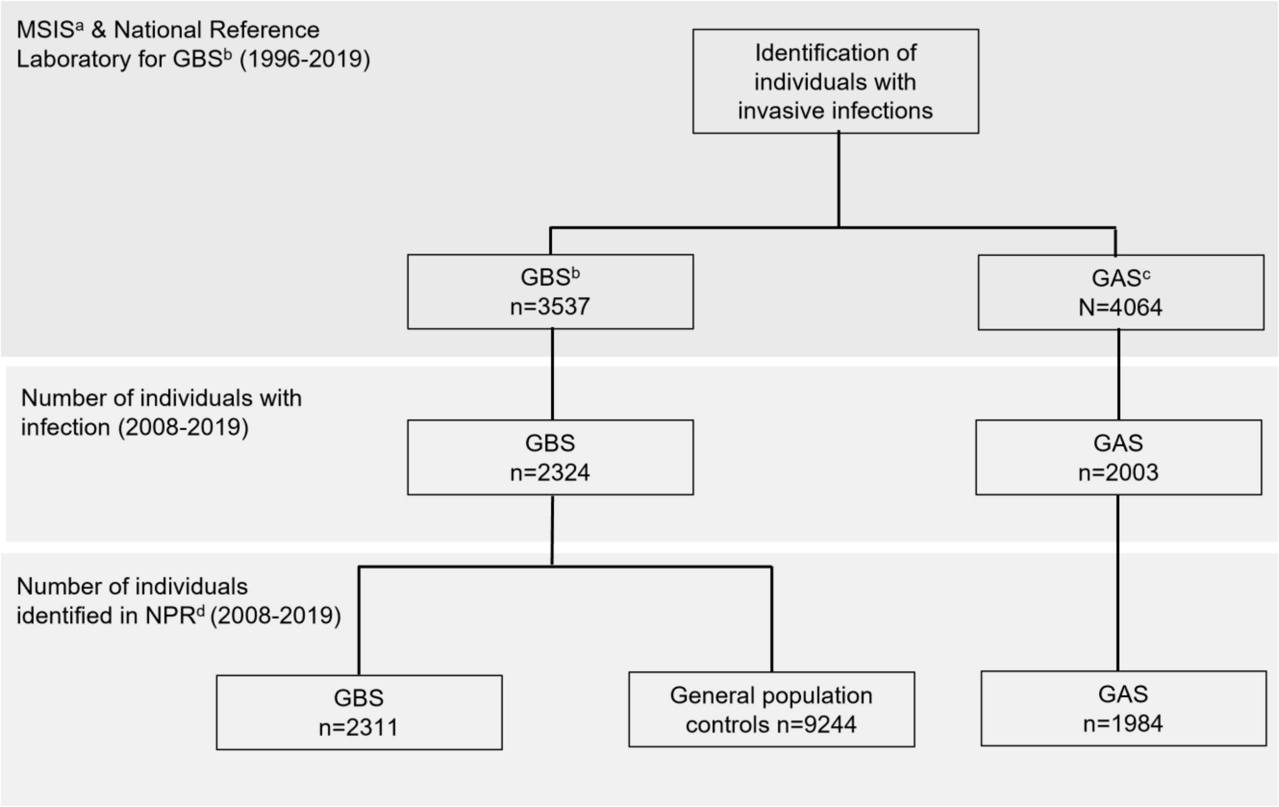

Supplementary table 1: Age standardized incidence rates per 100 000 person years with 95% confidence intervals (95CI), for A: invasive group B streptococcal disease[a] & B: invasive group A streptococcal disease

A: Invasive group B streptococcal disease					B: Invasive group A streptococcal disease				
Year	N	Crude rate	Adjusted rate	95% CI	Year	N	Crude rate	Adjusted rate	95CI
1996	3,357,033	1.10	1.10	0.80-1.50	1996	3,357,033	2.40	2.40	1.90-2.90
1997	3,370,423	1.20	1.30	0.90-1.70	1997	3,370,423	4.20	4.20	3.50-4.90
1998	3,386,063	1.30	1.30	0.90-1.60	1998	3,386,063	6.80	6.90	6.00-7.80
1999	3,405,060	2.60	2.50	2.00-3.10	1999	3,405,060	6.80	6.80	5.90-7.70
2000	3,425,653	2.50	2.50	1.90-3.00	2000	3,425,653	4.50	4.50	3.80-5.20
2001	3,442,579	2.60	2.60	2.00-3.10	2001	3,442,579	5.40	5.40	4.60-6.20
2002	3,456,577	2.60	2.60	2.10-3.20	2002	3,456,577	3.80	3.80	3.10-4.40
2003	3,476,541	2.90	2.90	2.30-3.40	2003	3,476,541	5.50	5.60	4.80-6.40
2004	3,495,131	3.30	3.30	2.70-4.00	2004	3,495,131	6.20	6.20	5.40-7.00
2005	3,518,330	3.60	3.50	2.90-4.10	2005	3,518,330	6.70	6.70	5.90-7.60
2006	3,547,491	3.90	3.80	3.20-4.50	2006	3,547,491	4.00	4.00	3.30-4.70
2007	3,585,131	4.20	4.20	3.50-4.90	2007	3,585,131	3.30	3.30	2.70-3.90
2008	3,637,892	3.70	3.60	3.00-4.30	2008	3,637,892	4.30	4.20	3.50-4.90
2009	3,695,771	3.90	3.90	3.30-4.50	2009	3,695,771	4.00	3.90	3.30-4.60
2010	3,749,043	3.90	3.80	3.20-4.50	2010	3,749,043	3.70	3.70	3.10-4.30
2011	3,805,931	4.30	4.30	3.60-5.00	2011	3,805,931	4.00	4.10	3.40-4.70
2012	3,867,645	4.60	4.60	3.90-5.30	2012	3,867,645	3.20	3.10	2.60-3.70
2013	3,928,378	4.60	4.60	3.90-5.30	2013	3,928,378	4.30	4.30	3.60-4.90
2014	3,983,895	4.80	4.90	4.20-5.50	2014	3,983,895	4.10	4.10	3.50-4.80
2015	4,040,198	5.30	5.30	4.60-6.10	2015	4,040,198	4.50	4.50	3.80-5.10
2016	4,086,583	5.50	5.50	4.70-6.20	2016	4,086,583	4.10	4.10	3.50-4.70
2017	4,127,266	5.00	4.90	4.20-5.60	2017	4,127,266	5.20	5.10	4.50-5.80
2018	4,166,612	5.90	5.70	5.00-6.50	2018	4,166,612	4.70	4.60	4.00-5.30
2019	4,205,704	7.00	6.70	5.90-7.50	2019	4,205,704	4.60	4.60	3.90-5.20

[a]Available data on age in 3522 (99.6%) of GBS patients

Supplementary table 2: Incidence rate (IR) per 100 00 person years by age group for invasive group B streptococcal disease

Variable	Age 18-39 n= 505		Age 40-59 n= 660		Age 60-79 n= 1518		Age 80+ n= 839	
Year	No. of cases	IR (95%CI)	No. of cases	IR (95CI)	No. of cases	IR (95CI)	No. of cases	IR (95CI)
1996	11	1.13 (0.62-2.03)	10	1.59 (0.88-2.86)	16	1.59 (0.88-2.86)	8	4.53 (2.27-9.06)
1997	11	1.73 (0.96-3.12)	10	2.47 (1.54-3.98)	26	2.47 (1.54-3.98)	5	2.77 (1.15-6.66)
1998	15	1.16 (0.70-1.92)	9	1.76 (1.00-3.10)	15	1.76 (1.00-3.10)	8	4.34 (2.17-8.67)
1999	21	1.62 (1.06-2.49)	18	4.43 (3.10-6.34)	40	4.43 (3.10-6.34)	18	9.55 (6.02-15.16)
2000	29	2.16 (1.50-3.10)	13	3.70 (2.50-5.48)	36	3.70 (2.50-5.48)	18	9.47 (5.97-15.03)
2001	22	3.21 (2.11-4.87)	22	3.43 (2.28-5.16)	29	3.43 (2.28-5.16)	22	11.17 (7.36-16.97)
2002	22	1.59 (1.04-2.41)	16	4.65 (3.27-6.62)	42	4.65 (3.27-6.62)	22	10.92 (7.19-16.58)
2003	21	1.64 (1.07-2.52)	28	2.30 (1.58-3.32)	36	4.04 (2.77-5.90)	24	11.70 (7.84-17.46)
2004	14	1.43 (0.85-2.41)	32	2.59 (1.83-3.67)	56	6.23 (4.60-8.43)	29	13.86 (9.63-19.95)
2005	23	1.82 (1.21-2.75)	30	2.41 (1.68-3.44)	49	5.81 (4.26-7.92)	32	15.01 (10.62-21.23)
2006	28	2.72 (1.88-3.94)	27	2.15 (1.47-3.13)	57	6.80 (5.13-9.03)	34	15.75 (11.25-22.04)
2007	19	1.45 (0.92-2.27)	25	1.98 (1.34-2.92)	68	9.05 (7.11-11.52)	39	17.88 (13.06.24.47)
2008	24	1.81 (1.21-2.70)	21	1.64 (1.07-2.52)	49	6.52 (4.92-8.62)	40	18.30 (13.42-24.95)
2009	18	1.41 (0.89-2.23)	36	2.78 (2.00-3.85)	57	7.38 (5.69-9.56)	32	14.57 (10.30-20.60)
2010	15	1.16 (0.70-1.93)	32	2.43 (1.72-3.44)	48	6.05 (4.56-8.02)	50	22.73 (17.22-29.98)
2011	16	1.66 (1.02-2.71)	32	2.40 (1.70-3.40)	75	9.22 (7.35-11.56)	40	18.09 (13.27-24.66)
2012	17	1.28 (0.79-2.06)	37	2.73 (1.98-3.77)	81	9.73 (7.82-12.10)	42	18.95 (14.01-25.65)
2013	22	1.48 (0.98-2.26)	31	2.26 (1.59-3.21)	82	9.61 (7.74-11.93)	46	20.76 (15.55-27.72)
2014	28	1.95 (1.35-2.83)	36	2.60 (1.87-3.60)	88	10.03 (8.14-12.36)	39	17.67 (12.91-24.18)
2015	31	2.13 (1.50-3.03)	34	2.43 (1.74-3.40)	104	11.54 (9.52-13.98)	46	20.87 (15.63-27.86)
2016	29	2.62 (1.82-3.77)	40	2.84 (2.08-3.87)	105	11.34 (9.36-13.73)	51	23.18 (17.62-30.50)
2017	19	1.29 (0.82-2.02)	34	2.41 (1.72-3.37)	94	9.88 (8.07-12.10)	58	26.25 (20.29-33.95)
2018	24	1.69 (1.13-2.52)	34	2.40 (1.72-3.36)	121	12.42 (10.40-14.85)	66	29.63 (23.28-37.71)
2019	26	1.73 (1.18-2.55)	53	3.73 (2.85-4.88)	144	14.45 (12.27-17.01)	70	30.97 (24.50-39.15)

Supplementary table 3: Incidence rates (IR) per 100 000 person-years of invasive group b streptococcal disease (GBS) according to year and sex and incidence rate ratios (IRR) comparing men and women overall and within age groups

	IR (95% CI)		IRR (95% CI) comparing men vs women				
Year	Men	Women	Overall	18-39 years	40-59 years	60-79 years	≥ 80 years
1996-2000	1.57 (1.30-1.90)	2.25 (1.95-2.60)	0.70 (0.55-0.88)	0.17 (0.09-0.34)	0.76 (0.45-1.29)	1.07 (0.72-1.60)	1.95 (1.16-3.28)
2001-2005	2.86 (2.50-3.27)	3.58 (3.21-4.00)	0.80 (0.70-0.95)	0.28 (0.17-0.47)	0.75 (0.53-1.06)	1.16 (0.85-1.58)	1.51 (1.07-2.14)
2006-2010	4.11 (3.70-4.57)	3.98 (3.59-4.42)	1.03 (0.89-1.20)	0.31 (0.2-0.49)	0.97 (0.70-1.35)	1.66 (1.30-2.12)	1.71 (1.29-2.27)
2011-2015	5.43 (4.98-5.93)	4.41 (4.02-4.84)	1.23 (1.08-1.4)	0.28 (0.18-0.44)	1.02 (0.76-1.38)	1.86 (1.53-2.27)	2.06 (1.57-2.69)
2016-2019	6.79 (6.24-7.40)	5.41 (4.92-5.94)	1.26 (1.11-1.43)	0.32 (0.20-0.51)	1.06 (0.78-1.45)	1.76 (1.45-2.12)	1.88 (1.46-2.41)

Abbreviations: CI = confidence interval

Supplementary fig. 2: Number of unique patients where aerobic blood cultures were drawn, from selected hospitals during the study period 1996-2019

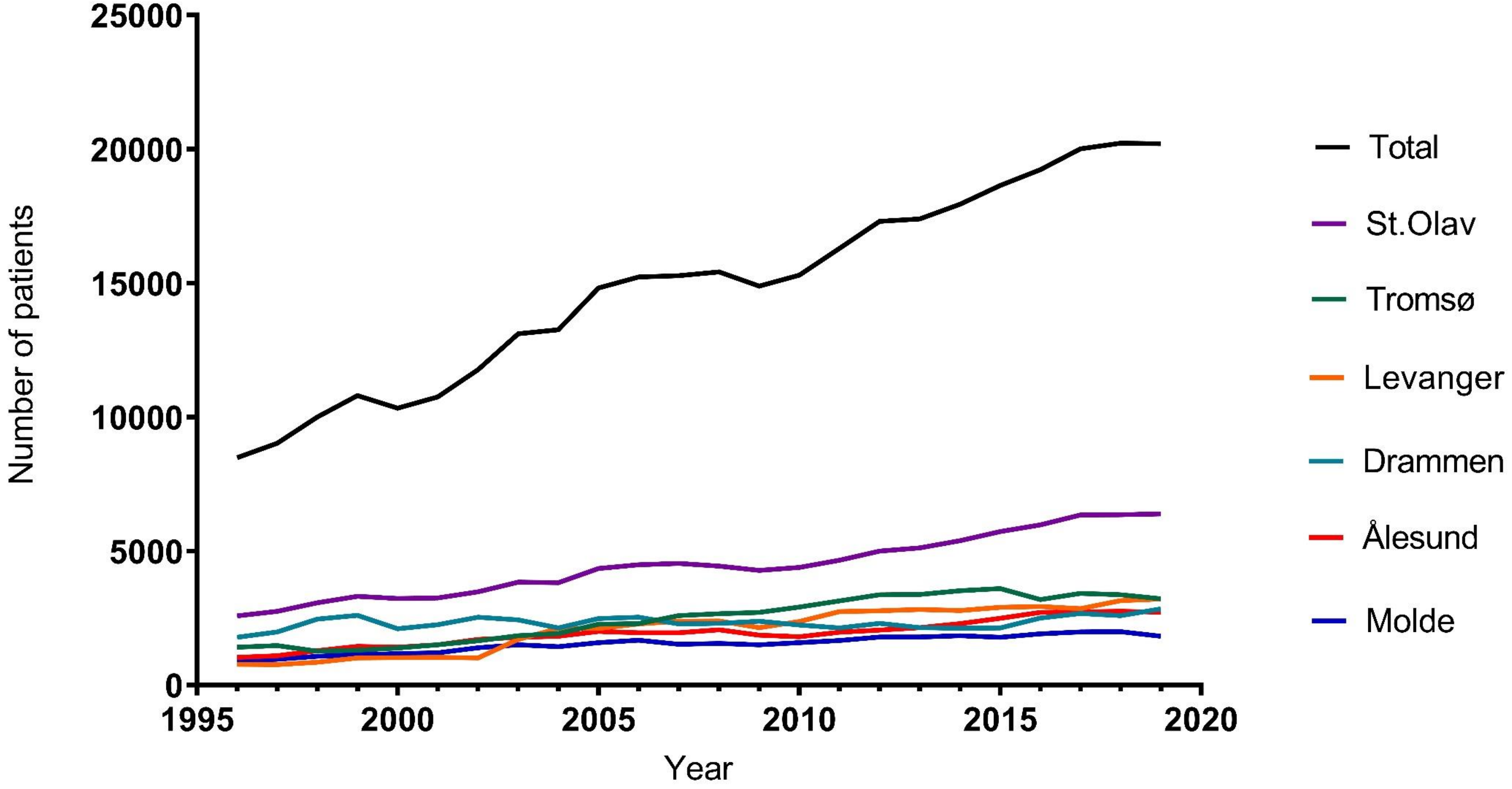

Supplementary table 4: Number of aerobic blood cultures and unique patients sampled, from selected hospitals during the study period 1996-2019

Hospital	University hospital of Tromsø		Drammen hospital		Levanger hospital		St. Olavs hospital		Ålesund hospital		Molde hospital	
Year	Blood cultures	Patients	Blood cultures	Patients	Blood cultures	Patients	Blood cultures	Patients	Blood cultures	Patients	Blood cultures	Patients
1996	6715	1412	5264	1783	1246	773	7619	2581	3287	1033	2704	901
1997	6805	1475	6102	1980	1194	765	8022	2754	3472	1091	2819	963
1998	6482	1259	7388	2461	1346	851	8984	3073	3706	1283	3315	1074
1999	6538	1299	8456	2603	1600	1012	9205	3312	4261	1446	3645	1131
2000	7027	1387	7201	2104	1543	1025	8829	3233	3902	1418	3521	1169
2001	7598	1502	7758	2255	1566	1037	8960	3250	4367	1500	3623	1205
2002	6399	1654	9047	2528	1495	1012	10211	3479	5314	1704	4409	1398
2003	5478	1839	9173	2430	3262	1707	11089	3843	5646	1783	4521	1505
2004	6027	1927	7809	2134	4408	2114	11374	3822	5710	1820	4485	1438
2005	6811	2271	8971	2486	4525	2112	12783	4354	6162	2007	5021	1588
2006	6979	2293	9074	2535	5101	2283	13701	4494	6250	1954	5121	1668
2007	11453	2587	8483	2285	4101	2386	13584	4538	6075	1957	4861	1528
2008	13991	2666	8375	2306	4087	2394	13549	4437	6566	2058	5053	1555
2009	13522	2713	8145	2384	3543	2144	12434	4279	5808	1864	4901	1503
2010	15534	2912	6690	2239	4236	2368	12548	4394	5682	1800	5082	1584
2011	16450	3140	6535	2133	4966	2737	13446	4657	6619	1974	5300	1659
2012	18680	3372	7437	2307	4979	2776	14756	5002	6393	2055	5615	1790
2013	18510	3384	6950	2139	5019	2819	15059	5121	6515	2138	5547	1793
2014	20458	3523	7295	2124	4944	2783	15805	5384	7119	2289	5764	1843
2015	11229	3600	7130	2130	5520	2903	16946	5732	8154	2492	5789	1786
2016	11836	3191	8359	2503	8333	2934	17272	5976	8244	2708	6093	1915
2017	11849	3418	9213	2671	7976	2850	18858	6348	7693	2733	6428	1986
2018	12258	3375	9741	2594	9124	3153	19139	6356	8057	2750	6500	1989
2019	12017	3220	10711	2843	9178	3212	18684	6388	8645	2718	5759	1822

Supplementary table 5: Proportion of patients with invasive group B streptococcus (GBS) infections who had selected comorbid conditions and Odds ratios compared to patients with invasive group a streptococcus (GAS) infections and general population controls

**Control group**		**GBS**	**GBS/GAS**	**Adjusted GBS/GAS [a]**	**GBS/General population**	**Adjusted GBS/ General population [a]**
**Variable:**	No of cases	% [95% CI]	OR [95%CI]	OR [95%CI]	OR [95%CI]	OR [95%CI]
**Cancer**	592	25.6 (23.8-27.4)	2.33	2.13 (1.81-2.51)	3.86	3.79 (3.21-4.48)
Period 2008-2013	187	19.8 (17.4-22.5)	2.30	2.01 (1.59-2.77)	4.19	4.17 (3.09-5.63)
Period 2014-2019	405	29.5 (27.1-31.9)	2.31	2.13 (1.74-2.60)	3.70	3.64 (2.98-4.44)
**Cardiovascular disease**	1028	44.4 (42.4-46.4)	1.93	1.62 (1.40-1.86)	3.37	3.36 (2.91-3.89)
Period 2008-2013	357	37.9 (34.8-41.0)	1.85	1.56 (1.26-1.94)	3.64	4.02 (3.17-5.09)
Period 2014-2019	671	48.8 (46.2-51.5)	1.96	1.63 (1.36-1.96)	3.21	3.01 (2.51-3.62)
**Respiratory disease**	257	11.1 (9.9-12.4)	1.23	1.11 (0.90-1.35)	1.66	1.75 (1.43-2.14)
Period 2008-2013	84	8.9 (7.3-10.9)	1.37	1.25 (0.88-1.78)	1.96	1.94 (1.34-2.82)
Period 2014-2019	173	12.6 (10.9-14.5)	1.96	1.02 (0.80-1.31)	1.53	1.68 (1.32-2.13)
**Gastrointestinal disease**	110	4.8 (4.0-5.7)	1.36	1.43 (1.05-1.95)	3.82	3.32 (2.37-4.67)
Period 2008-2013	39	4.1 (3.0-5.6)	1.87	2.07 (1.19-3.60)	4.98	4.68 (2.54-8.61)
Period 2014-2019	71	5.2 (4.1-6.5)	1.24	1.19 (0.82-1.72)	3.40	2.86 (1.88-4.32)
**Reumatic disease**	182	7.9 (6.8-9.0)	1.16	1.03 (0.82-1.31)	1.92	1.83 (1.44-2.34)
Period 2008-2013	58	6.2 (4.8-7.9)	1.03	0.91 (0.61-1.34)	2.56	2.16 (1.33-3.52)
Period 2014-2019	124	9.0 (7.6-10.7)	1.22	1.09 (0.81-1.47)	1.71	1.79 (1.32-2.32)
**Urogenital disease**	370	16.0 (14.5-17.5)	1.85	1.59 (1.31-1.92)	5.02	5.50 (4.43-6.83)
Period 2008-2013	105	11.1 (9.3-13.3)	1.69	1.51 (1.08-2.11)	5.08	5.50 (3.65-8.29)
Period 2014-2019	265	19.3 (17.3-21.5)	1.88	1.60 (1.26-2.02)	5.00	5.51 (4.27-7.11)
**Skin & soft tissue disease**	77	3.3 (2.7-4.1)	1.13	1.09 (0.77-1.55)	2.11	2.34 (1.65-3.31)
Period 2008-2013	23	2.4 (1.6-3.7)	1.68	1.55 (0.78-3.10)	3.22	3.01 (1.52-5.98)
Period 2014-2019	54	3.9 (3.0-5.1)	0.95	0.94 (0.62-1.40)	1.83	2.15 (1.43-3.22)
**Thyreotoxicosis**	20	0.9 (0.6-1.3)	1.58	1.60 (0.76-3.38)	1.51	2.21 (1.14-4.28)
Period 2008-2013	4	0.4 (0.2-1.1)	0.94	0.96 (0.23-3.94)	1.33	1.40 (0.16-12.03)
Period 2014-2019	16	1.2 (0.7-1.9)	1.87	1.90 (0.77-4.66)	1.56	2.38 (1.18-4.78)

**Neurological disease**	176	7.6 (6.6-8.8)	1.65	1.57 (1.21-2.04)	2.40	2.58 (2.03-3.30)
Period 2008-2013	54	1.1 (1.0-1.3)	1.62	1.60 (1.02-2.52)	3.76	4.27 (2.69-6.78)
Period 2014-2019	122	2.3 (2.1-2.6)	1.63	1.52 (1.11-2.09)	2.05	2.14 (1.60-2.86)

[a]Adjusted for age and gender

Supplementary table 6: Capsular serotype (CPS) and alpha-like surface protein (Alp)[a] genes in 3423 strains of group B streptococcus (GBS) isolated from adults with invasive infection in Norway, presented in 5-year periods.

CPS and Alp genes	Proportion of GBS strains with CPS and Alp genes, with 95% CI				
	**1996 -2000**	**2001-2005**	**2006-2010**	**2011-2015**	**2016-2019**
**CPS genes**					
Ia	20.5 (14.8-27.9)	14.6 (10.9-19.2)	16.2 (13.5-19.3)	15.0 (12.8-17.5)	17.9 (15.7-20.5)
Ib	8.9 (5.2-14.7)	10.3 (7.3-14.5)	6.8 (9.4-13.5)	11.3 (9.4-12.5)	10.9 (9.1-13.0)
II	9.6 (5.8-15.5)	10.3 (7.3-14.5)	10.7 (8.5-13.4)	14.0 (22.9-26.5)	13.4 (11.4-15.7)
III	24.0 (17.7-31.6)	21.0 (16.6-26.2)	19.1 (16.2-22.4)	18.7 (16.3-21.3)	18.9 (16.5-21.5)
IV	2.7 (1.0-7.1)	11.7 (8.5-16.1)	16.5 (13.8-19.6)	12.3 (10.3-14.6)	11.7 (9.8-13.9)
V	32.9 (25.7-40.9)	28.8 (23.8-34.4)	28.8 (25.4-32.5)	24.1 (24.4-27.0)	21.3 (18.9-24.0)
Other[b]	1.4 (0.3-5.4)	0.4 (0.1-2.5)	1.9 (1.1-3.4)	4.2 (3.1-5.7)	5.9 (4.5-7.5)
**Alp genes**					
alp2/3	30.8 (23.8-38.8)	27.8 (22.8-33.3)	26.4 (23.1-30.0)	23.8 (21.2-26.8)	21.5 (19.0-24.2)
bca	20.6 (14.7-22.9)	20.3 (16.0-25.4)	15.7 (13.1-18.8)	27.0 (24.2-30.0)	27.1 (24.4-30.0)
epsilon	20.6 (14.7-27.9)	25.6 (20.8-31.1)	33.1 (29.5-36.9)	25.1 (22.4-28.0)	24.5 (21.9-27.3)
rib	25.3 (18.9-33.1)	21.7 (17.3-26.9)	22.5 (19.4-26.0)	22.8 (20.1-25.6)	25.1 (22.5-27.9)
NT[c]	2.7 (1.0-7.1)	4.6 (2.7-7.8)	2.3 (1.4-3.8)	1.3 (0.8-2.3)	1.9 (1.2-2.9)

[a]Surface protein beta encoded by the *bac* gene was present in 565/3423 (16.4 %) GBS strains through the study period 1996-2019

bCPS types VI-IX and non-typable

cNon-typable

[d]Last period 2016-2019 is only four years
